# Identifying Gaps in the Treatment Guidelines for Hepatitis C in Peru to Meet International Standards: A Narrative Review

**DOI:** 10.3390/jcm13133867

**Published:** 2024-06-30

**Authors:** Jose A. Gonzales-Zamora, Carlos Quispe-Vicuña, Martín E. Reategui-Garcia, Julieta M. Araoz-Salinas, Fabricio Ccami-Bernal, Noelia Morocho-Alburqueque, Jian Pierre Espinoza-Herreros, Josue Layme, Gabriel Aquino-Sandoval, Victor Y. Melt Campos, Jorge Alave

**Affiliations:** 1 Division of Infectious Diseases, Department of Medicine, Miller School of Medicine, University of Miami, Miami, FL 33136, USA; jxg1416@med.miami.edu; 2 Peruvian American Medical Society (PAMS), Albuquerque, NM 87111, USA; julieta.araoz@unmsm.edu.pe (J.M.A.-S.); josue.layme@unmsm.edu.pe (J.L.); victor.campos@jax.ufl.edu (V.Y.M.C.); 3 Red de Eficacia Clínica y Sanitaria (REDECS), Lima 15048, Peru; vicunas998@gmail.com; 4 School of Medicine, Universidad Nacional de la Amazonía Peruana, Iquitos 16000, Peru; martin.reategui.031@unapiquitos.edu.pe (M.E.R.-G.); 20091c0329@unapiquitos.edu.pe (G.A.-S.); 5 School of Medicine, Universidad Nacional de San Agustín de Arequipa, Arequipa 04001, Peru; fabricamibernal@gmail.com; 6 School of Medicine, Universidad Nacional de Piura, Piura 200104, Peru; 0902017014@alumnos.unp.edu.pe; 7 School of Medicine, Universidad Privada Antenor Orrego, La Libertad 13006, Peru; pierre021996@gmail.com; 8 School of Medicine, Universidad Nacional Mayor de San Marcos, Lima 15001, Peru; 9 Sociedad Científica de Estudiantes de Medicina de la Amazonía Peruana (SOCIEMAP), Universidad Nacional de la Amazonía Peruana, Iquitos 16000, Peru; 10 Department of Community Health and Family Medicine, College of Medicine, University of Florida, Jacksonville, FL 32209, USA; 11 School of Medicine, Universidad Peruana Union, Lima 15464, Peru

**Keywords:** Hepatitis C, treatment, cirrhosis, renal disease, recommendations, Peru

## Abstract

Hepatitis C virus still represents a major cause of morbidity and mortality worldwide. In Peru, two national practice guidelines for the management of this infection were published more than 5 years ago; however, the latest breakthroughs in the treatment make it necessary to update these guidelines. We reviewed the most recent recommendations of the international guidelines and compared them with the current Peruvian guidelines. We found major differences, such as the use of Glecaprevir/Pibrentasvir as a first-line therapy, which is contemplated in the World Health Organization guideline, and recommended by American and European guidelines, but is not considered in the Peruvian guidelines. Another crucial difference lies in the management of patients with chronic kidney disease, who are treated nowadays with a variety of direct-acting antivirals, with no restrictions on the use of Sofosbuvir-based regimens in first-world countries, an approach that has not been adopted in Peru. We believe that standardization of the recommendations of the Peruvian guidelines is imperative, including the new therapeutic strategies that have emerged in recent years. We also suggest conducting a cost effectiveness analysis in the Peruvian context to allow for the implementation of new antivirals, and to achieve a better control of hepatitis C in the country.

## 1. Introduction

Globally, hepatitis C virus (HCV) infection continues to be a major cause of viral hepatitis-related morbidity and mortality. In recent years, it was estimated that more than 6 million people are affected by HCV [1] and it accounted for 17% of all viral hepatitis deaths [2]. Many of the cases are potentially preventable with timely and appropriate treatment. For this reason, the World Health Organization (WHO) established strategies to reach a reduction in new infections by 90% and deaths by 65% between 2016 and 2030 [3]. In the context of Peru, the management and understanding of this infection is crucial due to its impact on public health. According to estimates published in The Lancet Gastroenterology and Hepatology, the prevalence of HCV infection in Peru was 0.5%, affecting approximately 167,000 individuals within a range of 99,000 to 182,000 infected individuals [4]. However, it was shown that there was a higher prevalence in high-risk groups, such as hemodialysis patients [5]. Importantly, many of those affected were unaware of their condition until they developed advanced liver disease [6], underscoring the urgent need for improved detection and treatment of HCV infection to try to control the overall burden of the disease.

The Peruvian health system consists of two sectors: the public and the private system. For the provision of health services, the public sector is divided into the subsidized sector, managed by the Ministry of Health (MINSA), and the contributory sector, funded by employer’s contributions, known as Social Health Insurance (ESSALUD) [7]. Due to the high cost of hepatitis C medications, many patients choose to seek care at health facilities affiliated with MINSA or ESSALUD. Once diagnosed with hepatitis C at a health facility, patients are referred to a high-level hospital and categorized based on the severity of liver complications to prioritize treatment for those with documented liver damage according to the clinical practice guidelines (CPG). In Peru, there are different regimens recommended for hepatitis C; however, Glecaprevir/Pibrentasvir is not included as a first-line treatment, contrasting with the international guidelines. This regimen represents a major advance in the management of chronic hepatitis C, as the duration of treatment can be reduced to only 8 weeks. This change highlights the importance of keeping up to date with the most recent recommendations to improve clinical outcomes and reveals the need for defined and upgraded CPGs in Peru. Despite there being two CPGs in Peru, they are outdated as the Ministry of Health (MINSA) guideline [8] dates from 2018, and the Social Health Insurance (ESSALUD) guideline [9] was published in 2019. Neither recommends therapies with Sofosbuvir/Velpatasvir in patients with chronic kidney disease, contrasting with the new recommendations of international guidelines that recommend it as an option in this population [10]. In Peru, a medium-income country, this creates obstacles to translating scientific advances into a reduction in HCV morbidity. Well-designed programs encompassing prevention recommendations, screening, treatment, and updated strategic information are required [11]. Therefore, given the continuous evolution of treatment and eradication strategies for HCV, a critical analysis of the available clinical guidelines, and adaptation to the most appropriate treatment alternatives for the specific situation in Peru should be the cornerstone to updating the Hepatitis C management guidelines.

At approximately 5 years since the update of the last clinical practice guidelines for the treatment of HCV infection, it is imperative to summarize and compile the diagnostic and treatment measures that have been addressed around the world for a better management of patients with this disease in Peru, as this would contribute to improve the quality of life for all of them.

## 2. Methodology

In the present study, we conducted a narrative review of the main aspects of HCV treatment, and compared the recommendations contemplated in the national clinical practice guidelines of Peru (MINSA and ESSALUD) with those proposed in the international guidelines developed by the World Health Organization (WHO) [12], the American Association for the Study of Liver Diseases (AASLD)/Infectious Diseases Society of America (IDSA) [13], and the European Association for the Study of Liver (EASL) [14]. We only focused on the management of treatment-naive patients. We provided a succinct description of the most representative studies that supported the use of direct-acting antiviral drugs for HCV and included a table that summarized the recommendations of each guideline (Table 1). We finalized with a brief discussion about the cost-effectiveness of new hepatitis C antiviral regimens.

## 3. Main Aspects of Hepatitis C Treatment

### 3.1. Initiation of HCV Treatment

According to the MINSA guideline, all individuals with a confirmed diagnosis of HCV infection should have access to antiviral therapy; however, the treatment should be prioritized for certain groups, such as people with liver fibrosis stage F3 or F4, including those with decompensated cirrhosis; people with HIV or hepatitis B coinfection; patients with an indication for solid organ and tissue transplantation; people with recurrent HCV post-liver transplantation; those with significant extrahepatic manifestations (polyarteritis nodosa, neuropathy, cutaneous vasculitis, etc.); and individuals at risk of transmitting HCV (men who have sex with men, transgender females with high-risk sexual practices, patients on hemodialysis, etc.) [8]. On the other hand, the ESSALUD CPG reserves the treatment for people with fibrosis stage F2 and above, recommending only periodic evaluations according to clinical judgment and avoiding conditions that could exacerbate the progression of liver fibrosis for those with F0-F1 fibrosis [9]. For the evaluation of liver fibrosis, recommendations vary according to the type of health insurance. The MINSA guideline [8] mentions that this evaluation should be performed with non-invasive tests (APRI index and transitional elastography); however, the use of elastography is suggested only if available due to the shortage of resources in MINSA hospitals. On the other hand, the ESSALUD guideline [9] states that the evaluation of fibrosis should be performed with the APRI or FIB-4 index. Elastography is recommended only in patients in whom there is doubt about the results of the former tests. Hospitals from ESSALUD usually have enough resources for the performance of various tests, including elastography [15]. Liver biopsy is not considered in either of these two guidelines.

In recent years, many studies have shown significant benefits of treating HCV in the early fibrosis stages. In a study conducted by Jezequel et al., 820 patients with F0–F1 fibrosis were followed for 20 years, and it was shown that at 15 years, the survival rate was 92% in those with sustained virologic response (SVR), reaching 82% for those who had treatment failure, and 88% for those who were never treated. These differences were significant (*p* = 0.003) [16]. In addition, the modeling study published by Zahnd et al. found that delaying treatment for up to one year after diagnosis or until the development of F2, F3, or F4 fibrosis would result in an additional 13, 43, 142, and 418 cases of liver disease-related deaths, respectively, from 1000 HCV infections when compared to the strategy of treating all patients within one month of diagnosis. Concerning the time in which people are infectious, it increased from 5 years with early treatment (one month after diagnosis) to 21 years with late treatment for F4 fibrosis [17].

Currently, it is also known that HCV is not exclusively a hepatotropic pathogen and that HCV infection affects various metabolic functions. Therefore, treatment and elimination of the virus and of the inflammation secondary to HCV infection has had beneficial effects on the cardiovascular system and other organs [18]. This was demonstrated in the study by Adinolfi et al., in which the elimination of the virus by treatment with direct-acting antivirals reduced cardiovascular events by 2.0 to 3.5 times [19]. For these reasons, the international guidelines (WHO, AASLD/IDSA, EASL) recommend treatment for all patients with HCV regardless of the degree of fibrosis. Of note, the European guideline (EASL) proposes urgent Hepatitis C infection treatment in patients with significant fibrosis or cirrhosis (F2-F4), in people with extrahepatic manifestations, and in those at high risk of HCV progression or transmission [14].

### 3.2. Treatment of Patients without Cirrhosis or with Compensated Cirrhosis

Both the MINSA and ESSALUD guidelines exclusively recommend pan-genotypic regimens (with activity against genotypes 1–6) as first line, such as Velpatasvir/Sofosbuvir for 12 weeks. The utility of this regimen is based on several studies, among which the ASTRAL 1 clinical trial stands out. This study compared Velpatasvir/Sofosbuvir with a placebo for 12 weeks. It had a final sample of 740 patients with genotypes 1, 2, 4, or 6, of whom 19% had cirrhosis. Overall, the sustained virological response rate at 12 weeks post-treatment (SVR12) among patients receiving Velpatasvir/Sofosbuvir was 99%, while in the placebo group, no patient achieved SVR12. Of 121 patients with cirrhosis, 99% achieved SVR12 [20]. This great effectiveness was also corroborated in real-world studies, such as the one conducted by Mangia et al., which included 12 cohorts from North America and Europe, finding an effectiveness of 98.9% [21]. All of this makes Velpatasvir/Sofosbuvir unanimously considered a first-line treatment by the international guidelines (WHO, AASLD/IDSA, and EASL).

Another regimen mentioned only in the MINSA guideline as the first choice is Daclatasvir/sofosbuvir, which is a pan-genotypic regimen recommended for 12 weeks in patients without cirrhosis, extending the treatment duration to 24 weeks in patients with compensated cirrhosis. This regimen is still considered top-of-the-line in the WHO guideline. However, it is not recommended in the American or European guidelines given the availability of other more convenient and shorter treatment regimens in first-world countries.

One of the regimens that has revolutionized the management of hepatitis C is Glecaprevir/Pibrentasvir, a pan-genotypic antiviral that enables HCV cure in only 8 weeks. The scientific evidence for this regimen comes from several clinical trials and real-world studies. Regarding efficacy, one of the most important studies was conducted by Puoti et al., who made an integrated analysis of nine phase II and III clinical trials [22]. This study included 2041 patients without cirrhosis, and showed a very high cure rate, reaching an SVR12 of 98% with 8 weeks of Glecaprevir/Pibrentasvir, and 99% with 12 weeks; the difference between these two groups was not significant. Additionally, the subgroup analysis from patients with baseline characteristics traditionally associated with lower efficacy, such as previous treatment history, presence of NS3 or NS5A polymorphisms, race, high viral load, grade 3 fibrosis, etc., showed cure rates of more than 95%. In patients with compensated cirrhosis, the EXPEDITION-8 clinical trial revealed an SVR12 of 99% with Glecaprevir/Pibrentasvir for 8 weeks [23]. This high cure rate was also demonstrated in real-world studies. In this regard, Lampertico et al. conducted a meta-analysis of 18 cohorts and found an SVR12 of 96% in the intention-to-treat analysis and 98.1% in the modified analysis. Likewise, the good safety profile of this regimen was confirmed since adverse events occurred in 17.7% of patients, almost all of which were mild, leading to the discontinuation of the drug in only 0.6% of participants [24].

All this accumulation of evidence positions Glecaprevir/Pibrentasvir as a first-line drug for the treatment of patients with hepatitis C. According to the guidelines of the AASLD/IDSA and EASL, the duration of treatment for patients without cirrhosis or compensated cirrhosis is 8 weeks, making it the shortest treatment to date [13,14]. On the other hand, the WHO guideline recommends prolonging the treatment to 12 weeks in patients with compensated cirrhosis; however, it should be noted that the WHO guideline was published before the release of the EXPEDITION-8 trial results, a study that demonstrated the high efficacy of the 8-week regimen in patients with compensated cirrhosis.

Concerning treatments without pan-genotypic activity, the AASLD/IDSA guideline recommends Ledipasvir/Sofosbuvir for 8–12 weeks for genotypes 1, 4, 5, and 6; and Elbasvir/Grazoprevir for 12 weeks for genotypes 1 and 4 [13]. On the other hand, the EASL guideline proposes, in addition to Elbasvir/grazoprevir, the use of Sofosbuvir/Velpatasvir/Voxilaprevir for genotype 3 [14].

### 3.3. Treatment in Patients with Decompensated Cirrhosis

For this population, the MINSA guideline does not give specific recommendations on the drugs, it only mentions that the treatment should be carried out in highly complex health facilities, with experts involved in the management of these complications [8]. On the other hand, the ESSALUD guideline recommends the use of Velpatasvir/Sofosbuvir for 24 weeks or Velpatasvir/Sofosbuvir plus ribavirin for 12 weeks in adults with decompensated cirrhosis (Child-Pugh B or C), genotypes 1 to 6 [9]. This recommendation is in agreement with the European and American guidelines; however, the American guideline also considers Ledipasvir/Sofosbuvir for 24 weeks as a first-line regimen or for 12 weeks if used with ribavirin in patients with genotypes 1, 4, 5, and 6 [13]. This regimen has shown high efficacy, as demonstrated by the SOLAR-1 study, which revealed an SVR12 of 87% in patients with Child Turcotte Pugh (CTP) class B who received 12 weeks of treatment, and 89% in those who received 24 weeks of treatment [25]. In patients with CTP class C, the results were similar with the treatments of 12 and 24 weeks, obtaining an SVR12 of 86% and 87%, respectively. It should be noted that the frequency of serious adverse events increased with the treatment duration, reaching up to 34% in patients with CTP class C who received 24 weeks of treatment [25]. Most of these events were related to the use of ribavirin. Concerning the WHO guideline, it is briefly mentioned that the treatment of patients with decompensated cirrhosis should be carried out in centers with experience in the management of complications and with access to liver transplantation. In addition, it states that Daclatasvir, Velpatasvir, and Sofosbuvir have been studied in people with decompensated cirrhosis; however, no details are given about the specific regimens or treatment duration [12].

### 3.4. Treatment in Patients with Chronic Renal Disease

Regarding the treatment of patients with renal insufficiency, there are marked differences in the CPGs of Peru. While the MINSA CPG recommends Glecaprevir/Pibrentasvir for 8–12 weeks in treatment-naive patients, the ESSALUD CPG promotes the use of Grazoprevir/Elbasvir for 12 weeks. Currently, the treatment of patients with chronic kidney disease in developed countries does not differ from that used in the rest of the population, meaning that Glecaprevir/Pibrentasvir and Velpatasvir/Sofosbuvir are considered first-line therapies.

The use of Glecaprevir/Pibrentasvir in patients with chronic kidney disease is supported by two clinical trials: EXPEDITION-4 and EXPEDITION-5 [26,27]. The first one was an open-label, multicenter phase 3 trial that evaluated the efficacy and safety of this regimen for 12 weeks in patients with genotypes 1 to 6 with compensated liver disease (with or without cirrhosis) and grade 4/5 chronic kidney disease. It included patients on dialysis, which made up 82% of the population. The results showed an SVR12 of 98%, with no reports of virological failure [26]. A good safety profile was also noted, as no serious adverse events or grade 4 laboratory alterations directly related to the study drugs were described. The most common adverse events were pruritus, fatigue, and nausea [26]. On the other hand, 101 patients with chronic kidney disease were included in EXPEDITION-5 trial, 76% of whom were on dialysis. In contrast to EXPEDITION-4, in this study, 84% of participants received Glecaprevir/Pibrentasvir for 8 weeks, and despite this, 98% achieved SVR12, with no reports of virologic failure [26]. It is important to mention that the MINSA CPG stipulates treatment with Glecaprevir/Pibrentasvir for 12 weeks in patients with chronic renal disease and compensated cirrhosis; however, the current scientific evidence allows for an 8 week-treatment for this population as well. This has great advantages since reducing the duration of therapy can improve treatment adherence and reduce long-term adverse effects. Treatment longer than 8 weeks is only contemplated in the AASLD/IDSA and EASL guidelines for patients previously treated with ribavirin, interferon, or Sofosbuvir-based therapies [13,14].

Regarding Velpatasvir/Sofosbuvir, approximately 78% of the total elimination of Sofosbuvir’s main inactive metabolite, GS-331007, is through renal pathways [28]. A previous study showed that subjects with mild, moderate, and severe renal impairment had a 56%, 90%, and 456% higher GS-331007 area under the curve, respectively, compared to those with normal renal function [29]. Because of this, Sofosbuvir was not initially approved for HCV patients with severe or end-stage chronic kidney disease [30]. However, subsequent data reliably demonstrated the safety of this drug in patients with kidney disease, leading to the Food and Drug Administration’s (FDA) approval of Sofosbuvir-containing regimens in patients with chronic kidney disease and dialysis in 2019 [31]. Concerning Velpatasvir, its metabolism is mainly hepatic; therefore, its serum levels are not affected by the renal function. All this has made Velpatasvir/Sofosbuvir a first-line treatment for this population. The efficacy of this regimen was demonstrated in the study conducted by Borgia et al. in patients with end-stage chronic kidney disease on dialysis, reaching an SVR12 of 95% [32].

Although the AASLD/IDSA and EASL guidelines consider Velpatasvir/Sofosbuvir a first-line therapy in patients with chronic kidney disease, the WHO CPG does not recommend it; however, it should be noted that the WHO guideline dates back to 2018 when there was still no safety and efficacy data for Sofosbuvir-based regimens in this population [12]. Related to Glecaprevir/Pibrentasvir, it is recommended by the AASLD/IDSA and EASL guidelines as one of the therapies of choice. In the WHO guideline, although the efficacy and safety of the latter drug in patients with chronic kidney disease is recognized, it suggests considering other therapies due to the limited availability of Glecaprevir/Pibrentasvir in low- and middle-resource countries by 2018 [12].

Regarding Elbasvir/Grazoprevir, the ESSALUD guideline recommends it as first-line therapy in patients with chronic kidney disease; however, the main limitation of Elbasvir/Grazoprevir is that it is not a pan-genotypic regimen, being active only against genotypes 1 and 4. In addition, in patients with genotype 1a, it is necessary to evaluate the presence of resistance-associated substitutions (RASs) in NS5A because they have a significant impact on the cure rate, which can be reduced to up to 58% in the presence of these mutations [33]. Therefore, when NS5A RASs are detected, it is recommended to choose a different regimen or to prolong the treatment to 16–18 weeks and add ribavirin [34]. All this complicates the use of these medications, which are considered alternative therapies in first-world countries. In instances where genotyping is available, treatment with Elbasvir/Grazoprevir for 12 weeks is still recommended by the AASLD/IDSA guideline for genotype 1b and 4 in patients without cirrhosis or with compensated cirrhosis, and it is considered an alternative therapy in patients with genotype 1a [13]. According to the EASL guideline, Elbasvir/Grazoprevir should only be used in cases of genotype 1b [14]. Concerning the WHO guidance, this regimen is still recommended for genotypes 1 and 4 [12].

### 3.5. Treatment of HCV Post-Hepatic Transplant

For this population, there are no recommendations in the MINSA guideline; on the other hand, the ESSALUD guideline mentions a few recommendations for adult patients after liver transplantation with chronic HCV infection genotypes 1 to 6 without cirrhosis (F2 to F3) or with compensated cirrhosis (F4 and Child-Pugh A). This guideline suggests giving Sofosbuvir/Velpatasvir for 12 weeks or conducting individualized management of each case [9]. This recommendation is consistent with that outlined in the AASLD/IDSA and EASL guidelines; however, both guidelines also recommend Glecaprevir/Pibrentasvir, which is supported by data from the MAGELLAN-2 trial. This study evaluated a 12-week course of the daily fixed combination of Glecaprevir/Pibrentasvir in 80 liver transplant recipients and 20 kidney recipients without cirrhosis. It included patients with different HCV genotypes and showed an SVR12 rate of 98% [35].

Another regimen that has been considered in this population is Ledipasvir/Sofosbuvir plus ribavirin. Its use is based on the SOLAR-1 study, which included 223 liver transplant recipients with genotypes 1 or 4. The participants were randomly assigned to 12 or 24 weeks of treatment with a fixed combination of Ledipasvir/Sofosbuvir plus ribavirin. In patients without cirrhosis, an SVR12 rate of 96% and 98% was observed in the 12- and 24-week treatment arms, respectively. Efficacy was lower in patients with class B or C cirrhosis, with SVR12 rates of 86% and 88% in the 12- and 24-week arms, respectively [25]. Ledipasvir/Sofosbuvir +/− ribavirin is recommended only in the AASLD/IDSA guideline for this population but is not listed as a treatment of choice in the EASL guideline [13,14]. Regarding the WHO guideline, no recommendations are provided for post-liver transplant patients [12].

### 3.6. Treatment in Renal Transplant Recipients

The MINSA guideline does not provide recommendations for this population, while the ESSALUD guideline suggests administering Velpatasvir/Sofosbuvir for 12 weeks or individualized management for post-renal transplant adults without cirrhosis or compensated cirrhosis, which follows the current recommendations of the AASLD/IDSA and EASL. Another regimen that has been evaluated in this population is Glecaprevir/Pibrentasvir. In the MAGELLAN-2 study, 20 renal transplant recipients were included, and SVR12 was achieved in 100% [35]. For this reason, it is also included as a treatment of choice in the AASLD/IDSA and EASL guidelines. Unlike the EASL guideline, the AASLD/IDSA guideline additionally recommends Ledipasvir/Sofosbuvir in patients with HCV genotypes 1, 4, 5, and 6. This regimen has shown great efficacy in renal transplant recipients, even reaching an SVR12 of 100% in the clinical trial conducted by Colombo et al. [36]. Furthermore, the AASLD/IDSA guideline mentions Elbasvir/Grazoprevir as an alternative regimen solely for post-renal transplant patients with HCV genotypes 1 and 4 who do not have NS5A RAS mutations for Elbasvir [13]. Regarding the WHO guideline, no recommendations are provided for the treatment of this population.

## 4. Studies of Cost Effectiveness

Glecaprevir/Pibrentasvir has proven to be highly effective in treating HCV across all genotypes and diverse patient groups. Although not recommended by the Peruvian Guidelines, it is a first-line regimen for HCV according to all international guidelines (WHO, AASLD/IDSA, EASL). It is worth mentioning that several studies have also demonstrated its cost effectiveness. Among them, Kawaguchi et al. conducted a study in Japan in 2019 that evaluated the cost effectiveness of Glecaprevir/Pibrentasvir versus other direct-acting antivirals (DAAs) in chronic HCV. A health state transition model was developed to capture the natural history of HCV. Glecaprevir/Pibrentasvir was found to be dominant, generating higher-quality-adjusted life years (QALYs) and lower lifetime costs compared to all other DAAs. The predicted lifetime risk of hepatocellular carcinoma was lower for Glecaprevir/Pibrentasvir (3.66%) than for other DAAs (e.g., 4.99% for Elbasvir/Grazoprevir and 5.27% for Daclatasvir/Asunaprevir/Beclabuvir). The Glecaprevir/Pibrentasvir portfolio was cost-effective in 93.4% of simulations for a willingness-to-pay/QALY range of Japanese yen (JPY) of 1.6–20 million (USD 9,000–12,500). Glecaprevir/Pibrentasvir was a cost-effective strategy for genotype 1 treatment-naïve non-cirrhotic patients in Japan, and when considering a pan-genotypic framework, the Glecaprevir/Pibrentasvir portfolio outperformed Sofosbuvir-based options [37]. In Australia, another study showed that not only does the weighted published cost per course of Glecaprevir/Pibrentasvir generate an incremental cost-effectiveness ratio (ICER) that sits around the USD 15,000/QALY threshold set by the Pharmaceutical Benefits Advisory Committee (PBAC) for HCV drugs, the ICER was also well below the commonly used ICER threshold in other chronic diseases in Australia (USD 45,000/QALY). When the effective prices of Glecaprevir/Pibrentasvir were used, the ICERs sat well below the USD 15,000/QALY threshold used by PBAC for this therapy [38].

In Peru, one of the reasons why Glecaprevir/Pibrentasvir was not considered a first-line agent was its elevated cost. In this regard, the Institute of Evaluation of Technologies in Health and Investigation (IETSI) from ESSALUD conducted a budget impact analysis in 2018, revealing that the 12-week Glecaprevir/Pibrentasvir regimen’s price was PEN 101,814.32 (USD 27114.33), while the 8-week Glecaprevir/Pibrentasvir regimen’s cost was PEN 69,222 (USD 18,434.62). These prices exceeded those of other therapies at the time (Velpatasvir/Sofosbuvir and Daclatasvir/Sofosbuvir) [39], justifying the preference of alternative medications as first-line therapies. However, Glecaprevir/Pibrentasvir is currently included in the Pan American Health Organization (PAHO) Strategic Fund [40]; in addition, the Medicines Patent Pool [41] has already negotiated the manufacture of the generic form of this medication with four pharmaceutical companies, which will shortly lower the price of Glecaprevir/Pibrentasvir for low- and middle-income countries. Therefore, we believe a new cost effectiveness analysis should be conducted in Peru to reevaluate the use of this medication as a first-line therapy.

In terms of HCV treatment in patients with chronic renal disease, the IETSI in Peru conducted a budget impact analysis and concluded that the best therapy in this population was Elbasvir/Grazoprevir [39]. However, that analysis was performed in 2018, when Sofosbuvir-based therapies (e.g., Velpatasvir/Sofosbuvir) were not considered completely safe for patients with renal disease. Nowadays, new evidence has demonstrated the safety of these medications, which led to the approval of Velpatasvir/Sofosbuvir by the FDA for patients with chronic kidney disease and on dialysis [31]. According to the cost analysis conducted by IETSI at that time, the total implementation cost of Velpatasvir/Sofosbuvir was PEN 19,974.32 (USD 2656.28), lower than that of Elbasvir/Grazoprevir, which was PEN 36,072.57 (USD 9606.54) [39]. Based on these data, we believe that Sofosbuvir/Velpatasvir should be inserted as a first-line therapy for patients with chronic kidney disease and on dialysis.

## 5. Recommendations

After reviewing all the emerging data on HCV, we believe it is necessary to update the national Peruvian HCV guidelines and to harmonize the MINSA and ESSALUD recommendations, preferably adopting the most recent strategies contemplated in the international guidelines. We believe it is imperative to perform a periodic review of the scientific evidence to go hand in hand with the rapid progress of HCV treatment. One of the most important changes to consider would be the addition of Glecaprevir/Pibrentasvir as a first-line therapy, for which new cost-effectiveness studies should be conducted in Peru. We believe the availability of this medication should be examined to initiate negotiations with manufacturing companies in coordination with PAHO and the Peruvian government. Given the elevated prices of DAAs, a subsidy program should be considered to have broader access to these medications in Peru and to be able to start HCV treatment at the early stages of fibrosis in all individuals affected by this infection. Another important change we suggest for the Peruvian guidelines is to simplify the treatment of patients with chronic kidney disease by adding Velpatasvir/Sofosbuvir as a first-line regimen; a recommendation that is nowadays supported by many studies that confirm its high efficacy and excellent safety profile.

## 6. Conclusions

There are marked differences between Peru’s HCV clinical practice guidelines and the international guidelines, especially those developed in the United States and Europe, due to factors such as drug availability or logistical limitations. The main difference lies in the use of Glecaprevir/Pibrentasvir, which is considered the first line of treatment according to the WHO, AASLD/IDSA, and EASL guidelines, but it is not yet considered the first choice in the Peruvian guidelines. It is important to mention that since the publication of the most recent Peruvian guidelines, the progress in hepatitis C treatment worldwide has been enormous, with studies showing the high efficacy and excellent safety profile of Sofosbuvir-based regimens in patients with chronic kidney disease, which unfortunately has not been contemplated in the Peruvian guidelines. We believe that comparative studies like ours are crucial to identify gaps, strengths, and areas of improvement in clinical practices in low- and middle-income countries such as Peru. In this regard, we even found significant differences between the two Peruvian guidelines (ESSALUD and MINSA); therefore, we believe it is necessary to harmonize these two guidelines to facilitate HCV treatment nationally. Likewise, the global availability of new drugs makes it essential to update the treatment recommendations of Peruvian guidelines periodically, taking into account the new strategies mentioned by the international guidelines. These updates may allow for the approval, introduction and distribution of these medications in the country. We also suggest conducting cost effectiveness studies that are consistent with the reality of Peru and to implement public health measures to allow for broader access to these antivirals. Finally, this manuscript highlights the need to adapt the Peruvian guidelines according to international recommendations in order to provide an adequate management and treatment of hepatitis C, with the ultimate goal of preventing serious liver damage or cancer in patients affected by this condition and attempting the eradication of HCV in Peru in the medium or long term.

## Figures and Tables

**Table 1:** Main aspects of the HCV treatment according to the national guidelines of Peru and international guidelines.

Main Aspects of HCV Treatment	MINSA	ESSALUD	WHO	AASLD/IDSA	EASL
**Initiation of Treatment**	Treatment for all the patients with HCV but prioritized to certain groups: F3-F4 and population at high risk of progression	In patients with F2-F4 fibrosis	In all patients with HCV, regardless of the degree of fibrosis	In all patients with HCV, regardless of the degree of fibrosis	
**Treatment in patients without cirrhosis or with compensated cirrhosis**	Velpatasvir/Sofosbuvir for 12 weeks Daclatasvir/Sofosbuvir for 12 weeks	Velpatasvir/Sofosbuvir for 12 weeks	Velpatasvir/Sofosbuvir for 12 weeks Daclatasvir/Sofosbuvir for 12 weeks Glecaprevir/Pibrentasvir for 8–12 weeks	Velpatasvir/Sofosbuvir for 12 weeks Glecaprevir/Pibrentasvir for 8 weeks Ledipasvir/Sofosbuvir (only for genotypes 1, 4, 5, and 6) for 8–12 weeks Elbasvir/Grazoprevir (only for genotypes 1 and 4) for 12 weeks	Velpatasvir/Sofosbuvir for 12 weeks Glecaprevir/Pibrentasvir for 8 weeks Elbasvir/Grazoprevir (only for genotype 1b) for 12 weeks Sofosbuvir/Velpatasvir/Voxilaprevir (only for genotype 3 and compensated cirrhosis) for 12 weeks
**Treatment in patients with decompensated cirrhosis**	No recommendations are provided.	Velpatasvir/Sofosbuvir for 24 weeks Velpatasvir/Sofosbuvir + ribavirin for 12 weeks	No recommendations are provided	Velpatasvir/Sofosbuvir for 24 weeks Velpatasvir/Sofosbuvir + ribavirin for 12 weeks Ledipasvir/Sofosbuvir for 24 weeks (genotypes 1, 4, 5, and 6) Ledipasvir/Sofosbuvir + ribavirin for 12 weeks (genotypes 1, 4, 5, and 6)	Velpatasvir/Sofosbuvir for 24 weeks Velpatasvir/Sofosbuvir + ribavirin for 12 weeks
**Patients with chronic renal disease**	Glecaprevir/Pibrentasvir for 8–12 weeks	Elbasvir/Grazoprevir for 12 weeks (only for genotypes 1 y 4)	Elbasvir/Grazoprevir for 12 weeks (only for genotypes 1 and 4)	It is recommended to use the same regimens considered for patients without renal disease, including Sofosbuvir-based therapies	
**Treatment of HCV post-hepatic transplant**	No recommendations are provided.	Velpatasvir/Sofosbuvir for 12 weeks	No recommendations are provided	Velpatasvir/Sofosbuvir for 12 weeks Glecaprevir/Pibrentasvir for 12 weeks (not recommended in decompensated cirrhosis) Ledipasvir/Sofosbuvir +/- ribavirin for 12 weeks (genotypes 1, 4, 5, and 6)	Velpatasvir/Sofosbuvir for 12 weeks Glecaprevir/Pibrentasvir for 12 weeks (not recommended in decompensated cirrhosis)
**Treatment in renal transplant recipients**	No recommendations are provided.	Velpatasvir/Sofosbuvir for 12 weeks	No recommendations are provided	Velpatasvir/Sofosbuvir for 12 weeks Glecaprevir/Pibrentasvir for 12 weeks Ledipasvir/Sofosbuvir for 12 weeks (genotypes 1, 4, 5, and 6)	Velpatasvir/Sofosbuvir for 12 weeks Glecaprevir/Pibrentasvir for 12 weeks

## Data Availability

Not applicable.

## Abbreviations

MINSA: Ministry of Health of Peru; ESSALUD, Social Health Insurance; WHO, World Health Organization; AASLD, American Association for the Study of Liver Diseases; IDSA, Infectious Diseases Society of America; EASL, European Association for the Study of the Liver.
